# A case of needle tract seeding with endoscopic findings of progression over time

**DOI:** 10.1002/deo2.368

**Published:** 2024-04-21

**Authors:** Go Sawai, Katsuyuki Dainaka, Yoshida Juichiro, Yutaka Inada, Akifumi Fukui, Takeshi Nishimura, Hideki Fujii, Naoya Tomatsuri, Yusuke Okuyama, Hideki Sato

**Affiliations:** ^1^ Department of Gastroenterology and Hepatology Japanese Red Cross Society Kyoto Daiichi Hospital Kyoto Japan

**Keywords:** CA19‐9, endoscopic ultrasound fine‐needle aspiration, needle tract seeding, pancreatic cancer, submucosal tumor

## Abstract

An 83‐year‐old male underwent three transgastric punctures with endoscopic ultrasound‐guided fine‐needle aspiration for the examination of a pancreatic body tumor. After a diagnosis of resectable pancreatic cancer and undergoing distal pancreatectomy, the patient was administered postoperative adjuvant chemotherapy with oral S‐1 for 6 months, and carcinoembryonic antigen and carbohydrate antigen 19‐9 levels were bimonthly evaluated. Carbohydrate antigen 19‐9 levels continually increased to 4638.1 U/mL at 45 months post‐fine‐needle aspiration. Endoscopic ultrasound‐guided showed a 25 mm low‐echoic, irregularly shaped, and heterogeneous tumor with clear margins protruding from the mucosa outside the gastric wall, and biopsy confirmed adenocarcinoma. Since the immunostaining findings of the specimen matched those of the previously resected specimen, needle tract seeding (NTS) due to puncture of the pancreatic cancer was identified as the cause. After a pylorus‐preserving gastrectomy at 46 months post‐fine‐needle aspiration, postoperative chemotherapy initiation, comprising gemcitabine and nab‐paclitaxel, was initiated; however, the patient died despite these interventions as he developed multiple peritoneal dissemination. Although rare, the incidence of NTS will increase in the future owing to the expected extended survival in post‐pancreatic cancer resection cases. We suggest regular upper gastrointestinal endoscopy and endoscopic ultrasound‐guided evaluations for patients who are at risk for NTS can facilitate early detection. Furthermore, it is extremely relevant to share experiences of encountered NTS cases in practice and extend knowledge of its varying endoscopic appearances.

## INTRODUCTION

Endoscopic ultrasound‐guided fine‐needle aspiration (EUS‐FNA) is widely used for diagnosing pancreatic tumors histopathologically. EUS‐FNA is known for its safety and high diagnostic accuracy, with sensitivity and specificity of 89% and 96%,^1^ respectively. However, needle tract seeding (NTS) is a rare but significant complication of transgastric EUS‐FNA for pancreatic cancer. NTS involves the ectopic spread of tumor cells along the needle path during FNA biopsy and is particularly concerning in pancreatic body or tail cancers, a biopsy site often not included in the surgical resection area. In Japan, the reported incidence of NTS following EUS‐FNA for primary pancreatic cancer is 0.1%.^2^

Because chemotherapy is the first‐line treatment for unresectable and resectable cancers,^3^ the genetic analysis demand has increased, leading to multiple biopsy attempts to obtain adequate samples, thereby potentially increasing NTS incidence. No prospective studies have assessed the incidence and impact of NTS on prognosis, and there is no consensus on treatment approaches. The need for accumulating additional cases, statistically analyzing, and developing early detection and treatment methods is evident. This report shares our experience with NTS in pancreatic body cancer, focusing on the endoscopic findings and their chronological changes.

## CASE REPORT

**FIGURE 1:**
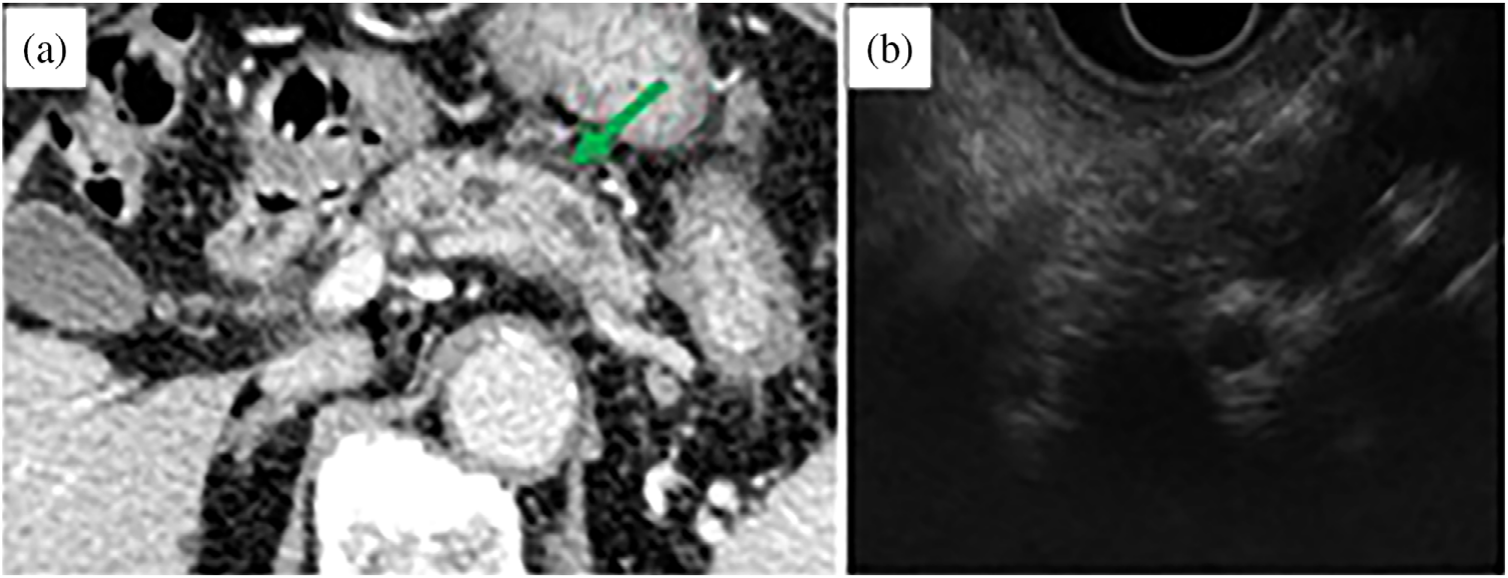
(a) The main pancreatic duct is disrupted in the pancreatic body, with a dilation of the distal main pancreatic duct. Contrast‐enhanced computed tomography shows a poor enhancement in the same region. (b) Endoscopic ultrasound shows an irregularly shaped hypoechoic mass, which is punctured.

An 83‐year‐old male with a history of hypertension and atrial fibrillation underwent a follow‐up abdominal contrast‐enhanced computed tomography (CT) scan after sigmoid colon cancer treatment. Scanning revealed a poorly enhanced lesion in the pancreatic body and the distal main pancreatic duct dilatation (Figure 1a).

EUS (GF‐UCT260; Olympus Medical Systems) revealed a 10 mm‐diameter low‐echoic tumor in the pancreatic body (Figure 1b). Three transgastric punctures were performed using a 22‐gauge Franseen needle (Acquire; Boston Scientific), employing the slow‐pull method. The histological diagnosis was an adenocarcinoma. Post‐procedural adverse events were absent.

Since the lesion was diagnosed as resectable pancreatic cancer, a distal pancreatectomy was performed (pT3pN0cM0, pStage IIA [UICC 8th]). Postoperative adjuvant chemotherapy with oral S‐1 (100 mg/day) was administered for 6 months, followed by bimonthly evaluation of carcinoembryonic antigen and carbohydrate antigen 19‐9 (CA19‐9) levels.

**FIGURE 2:**
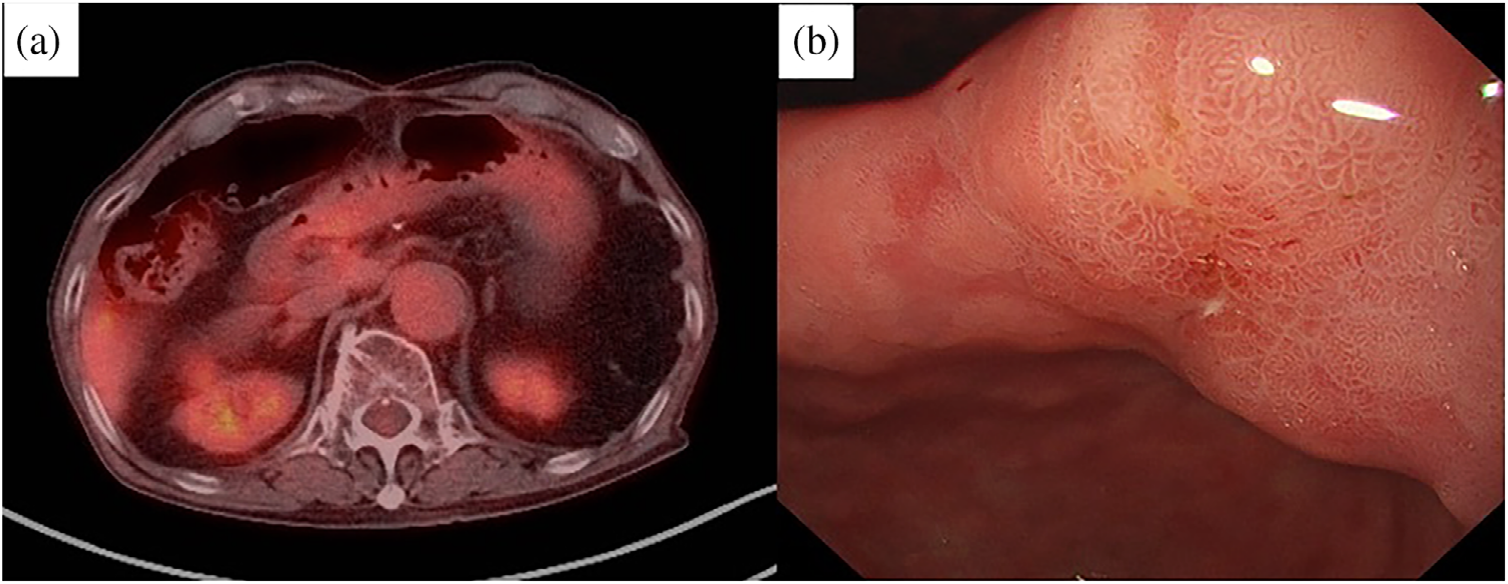
Forty‐one‐month post‐endoscopic ultrasound‐guided fine‐needle aspiration. (a) Positron emission tomography‐computed tomography shows no accumulation. (b) Upper gastrointestinal endoscopy reveals erosive lesions like ulcer scars on the lesser curvature of the gastric angle.

**FIGURE 3:**
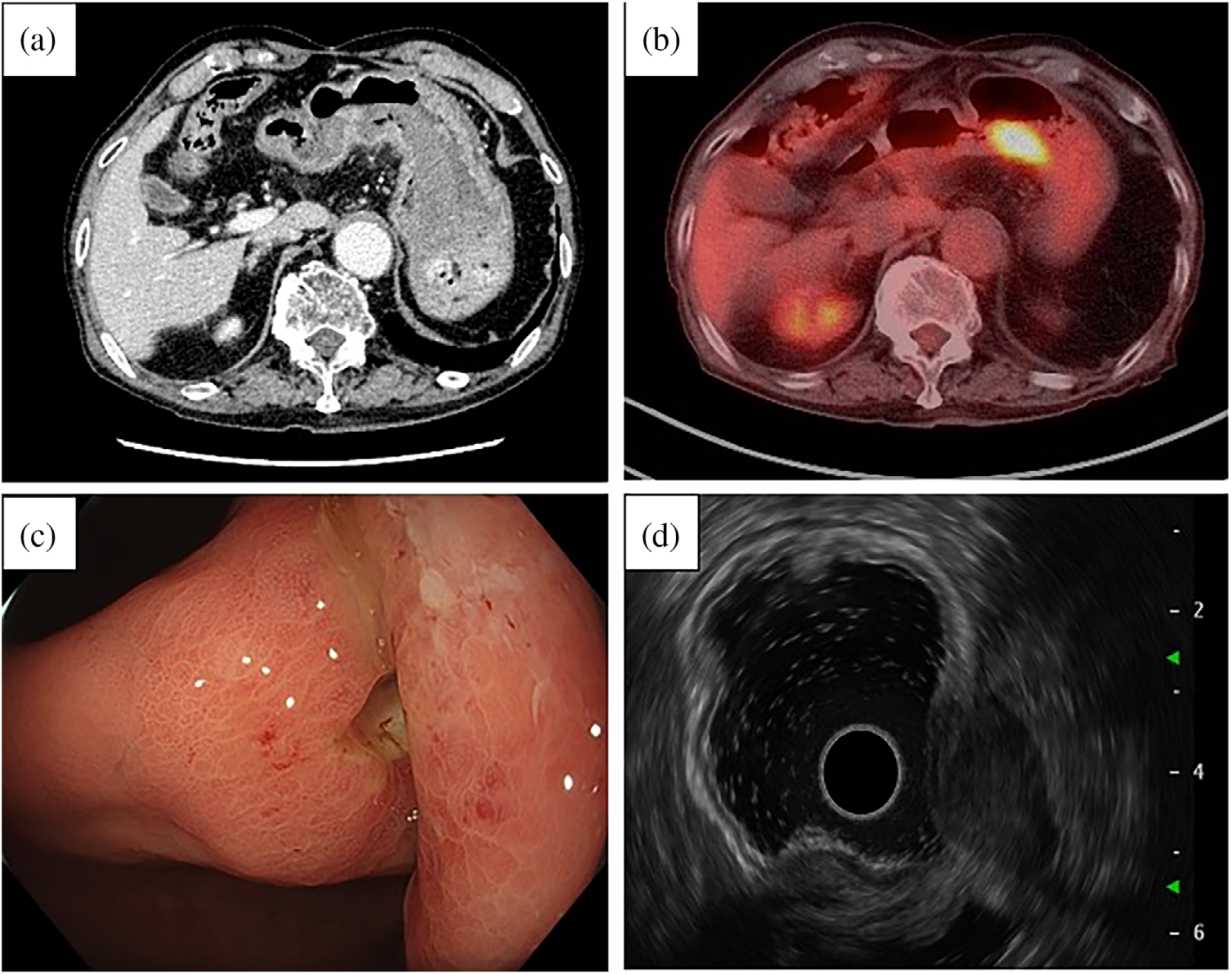
Forty‐five‐month post‐endoscopic ultrasound‐guided fine‐needle aspiration. (a) Contrast‐enhanced computed tomography shows no obvious thickening of the gastric wall or swelling of the surrounding lymph nodes. (b) The upper endoscopy reveals an ulcerative lesion at the same site as the previous biopsy. (c) Positron emission tomography‐computed tomography shows abnormal accumulation in the posterior wall of the gastric angle. (d) EUS shows a 25 mm low‐echoic, irregularly shaped, heterogeneous tumor with clear margins, protruding from the mucosa outside the gastric wall (white arrow).

At 37 months post‐FNA, the CA19‐9 levels increased to 145.2 U/mL; however, positron emission tomography‐CT (PET‐CT; Figure 2a) and ethoxybenzyl‐diethylenetriamenepentaacetic acid magnetic resonance imaging performed showed no evidence of cancer recurrence. At 41 months post‐FNA, upper gastrointestinal endoscopy revealed erosive lesions, such as ulcer scars, on the lesser curvature of the gastric angle, and biopsy disclosed gastritis (Figure 2b). However, 45 months post‐FNA, the CA19‐9 levels continually increased to 4638.1 U/mL. Though no findings suggestive of cancer recurrence were observed with contrast‐enhanced CT scan (Figure 3a), PET‐CT detected abnormal accumulation in the posterior wall of the gastric angle (Figure 3b), and upper endoscopy revealed an ulcerated lesion with sharply demarcated and raised margins at the same site as that in the previous biopsy (Figure 3c). EUS (GF‐UE290; Olympus Medical Systems) showed a 25 mm low‐echoic, irregularly shaped, and heterogeneous tumor with clear margins protruding from the mucosa outside the gastric wall (Figure 3d); biopsy of this lesion confirmed adenocarcinoma.

**FIGURE 4:**
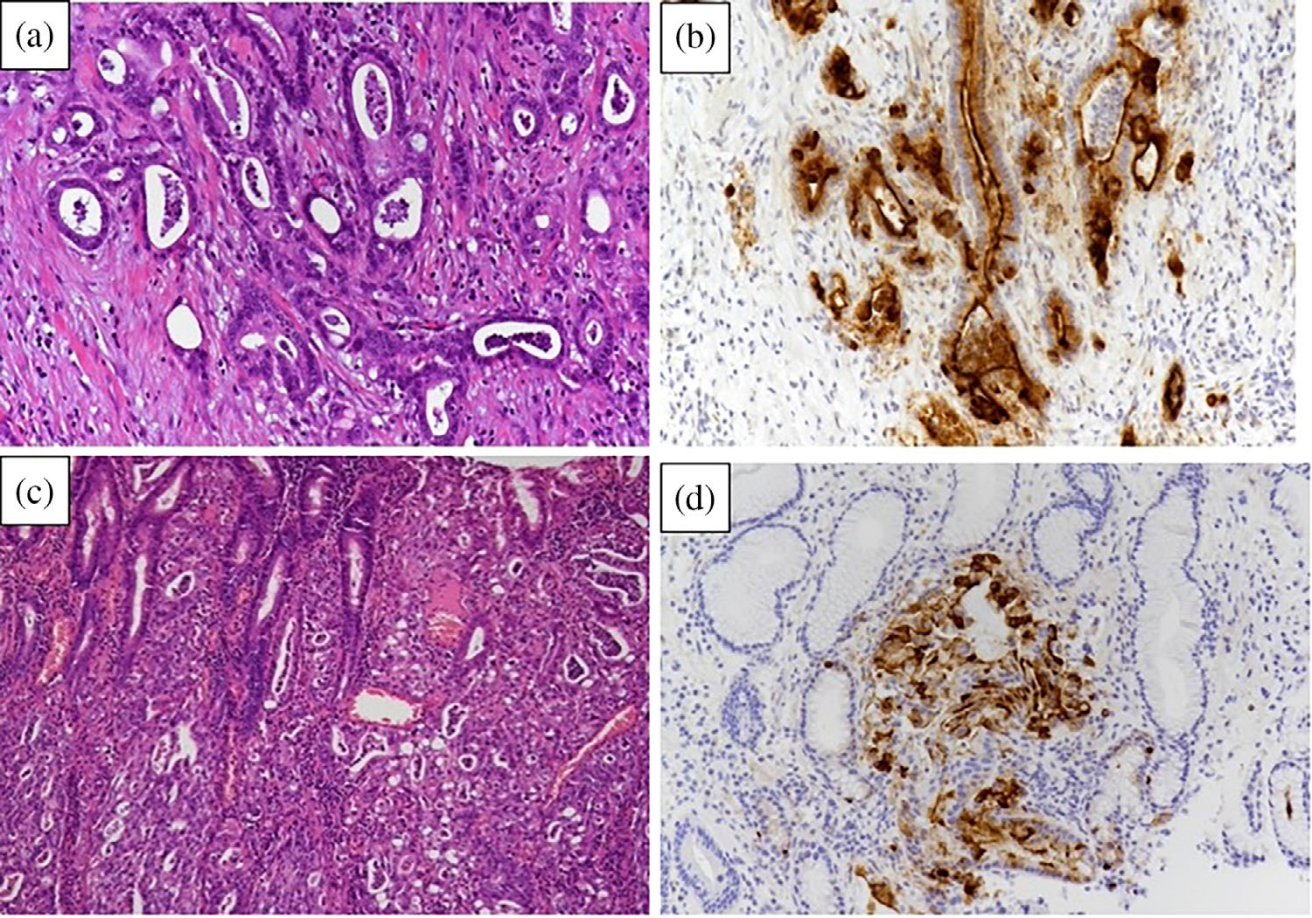
(a) Hematoxylin‐eosin‐stained of the pancreatic specimen at the distal pancreatectomy. The diagnosis is adenocarcinoma (por1 > tub2). (b) Staining of the pancreatic specimen with MUC1 at the same time as Figure 4a. (b) Hematoxylin‐eosin‐stained lesion of the biopsy tissue from the needle tract seeding lesion. Adenocarcinoma is distributed in all layers from the mucosa to the serosa, with no intraepithelial carcinoma component. (d) Mucin 1 stained lesion at the same time as Figure 4c.

The immunostaining findings of the specimen (MUC1(+), MUC2(‐), MUC5AC (+), MUC6(‐), MIB‐1 positive, and p53 completely negative) matched those of the previously resected specimen; therefore, we diagnosed NTS due to puncture of pancreatic cancer (Figure 4).

No evident metastases or recurrences were detected in other organs. At 46 months post‐FNA, a pylorus‐preserving gastrectomy was performed. Metastases were identified in several regional lymph nodes in the postoperative pathological diagnosis. Two months after surgery and 48 months post‐FNA, a PET‐CT scan indicated abnormal accumulation, suggestive of peritoneal dissemination, necessitating postoperative chemotherapy initiation, comprising gemcitabine at 720 mg/m^2^ and nab‐paclitaxel at 80 mg/m^2^. Considering the advanced age of the patient, the dose was adjusted to 70% of the standard regimen typically prescribed for postoperative recurrent pancreatic cancer. Despite these interventions, the patient developed multiple peritoneal disseminations and died 55 months post‐FNA.

## DISCUSSION

In recent years, the incidence of NTS after EUS‐FNA for pancreatic cancer has increased. The number of patients who underwent transgastric EUS‐FNA for pancreatic body or tail cancer is escalating with the rising importance of neoadjuvant chemotherapy for resectable pancreatic cancer and preoperative histological diagnosis after the Prep‐02/JSAP05 trial^4^ in 2019. NTS cases will increase in the future owing to the expected extended survival in post‐pancreatic cancer resection cases.

Given the rarity of NTS, additional case reports have to be accumulated for further studies. Kitano et al. reviewed this disease and reported a median duration of 19 months from puncture to NTS diagnosis.^5^ Furthermore, the period from FNA to diagnosis in this case was 45 months, a relatively long period before the NTS onset. Although the underlying mechanisms remain unclear, differences in the proliferation rates of tumor cells have been reported based on the presence of chemotherapy.^6^ This suggests that the histological type and sensitivity to neoadjuvant and adjuvant chemotherapies may influence the variability in the duration of NTS. Theoretically, a decrease in tumor cell volume due to chemotherapy may affect the time to diagnosis of NTS. In this case, adjuvant chemotherapy with oral S‐1 may have affected the time to NTS onset.

NTS often appears on the posterior wall of the gastric angle from the lower body, FNA puncture site. A typical submucosal tumor‐like lesion and occasionally diagnosed after gastrointestinal bleeding, NTS can lead to self‐destruction and ulcer formation.^5^ Endoscopic images taken at diagnosis did not show any evident elevated lesions in this case; contrarily, it progressed from an ulcer‐scar‐like lesion to the surrounding mound between 41 and 45 months after puncture, similar to the infiltrative ulcerative gastric cancer. We suggest that submucosal tumor elevations are common because seeding occurs in the submucosal layer, where the connective tissue is relatively loose. However, the histological type of pancreatic cancer and the amount of seeded tumor may lead to variations in the progression pattern, which could explain the variability in endoscopic images. In this case, we believe that only erosion was observed at the 41‐month post‐FNA endoscopic examination because the submucosal tumor had not grown vertically yet. 45‐month post‐FNA EUS findings of the protruded lesion outside the gastric wall suggest that a prominent part of the lesion is located outside the gastric wall; however, metastatic progression of the lesion extended it vertically towards the gastric wall. Additionally, this patient exhibited rapid endoscopic changes for 4 months. Although the NTS lesion presumably existed 41 months post‐FNA, the biopsy results were negative, and the lesion remained unidentified on PET‐CT. The endoscopic findings in this case show that NTS presentation on endoscopy varies, and EUS that allows observation beyond the submucosa may prove effective for early detection during the follow‐up of postoperative patients at NTS risk.

This case represents atypical endoscopic findings of NTS and captures its chronological endoscopic changes. Although an established treatment strategy for NTS is unavailable, reports have indicated favorable survival rates in patients undergoing local excision.^7^ Thus, therapeutic intervention at an early stage affects prognosis. We suggest regular upper gastrointestinal endoscopy and EUS evaluations for patients who are at risk for NTS can facilitate early detection.

## CONFLICT OF INTEREST STATEMENT

None.

## ETHICS STATEMENT

Not applicable.
